# Genomic Epidemiological Characteristics of Enteric Adenovirus F40 and F41 in Yantai, China, from 2022 to 2023

**DOI:** 10.3390/v17121595

**Published:** 2025-12-09

**Authors:** Peihua Niu, Zhenlu Sun, Yu Zhang, Dapeng Zhang, Yiming Zhao, Yi Zhang, Ping Cheng, Qiao Gao, Yu Li, Lili Zhao, Jie Yan, Hongmei Zheng, Xuejun Ma, Ji Wang

**Affiliations:** 1 National Key Laboratory of Intelligent Tracking and Forecasting for Infectious Diseases, NHC Key Laboratory of Medical Virology and Viral Diseases, National Institute for Viral Disease Control and Prevention, Chinese Center for Disease Control and Prevention, Beijing 102206, China; 2 Yantai Center for Disease Control and Prevention, Yantai 264003, China

**Keywords:** human adenovirus F subgroups, genomic epidemiology, single nucleotide variants, recombination, structural prediction

## Abstract

This study aims to investigate the genomic epidemiological characteristics of enteric adenovirus types F40 and F41 circulating in Yantai from August 2022 to August 2023, with a focus on genetic diversity, recombination events, and their potential implications for public health and vaccine development. A total of 1200 stool specimens were collected from patients with suspected viral diarrhea, and 47 human adenovirus F subgroup positive samples in Yantai were analyzed. HAdV-F subgroup nucleic acid detection and whole-genome sequencing were performed to identify virus types and single nucleotide variants (SNVs). Phylogenetic and recombination analyses were conducted, and viral protein structures were predicted using AlphaFold3. HAdV-F40 strains exhibited relative genetic conservation, with mutations primarily localized to the *fiber* gene. In contrast, HAdV-F41 strains displayed pronounced genetic divergence, with a higher mutation burden in the *hexon* gene. Recombination analysis revealed clear intra-typic recombination events within HAdV-F41, particularly in the *hexon* gene region. Structural modeling indicated that although the overall protein architecture remained intact, amino acid changes in key antigenic regions may influence immune evasion. HAdV-F40 and HAdV-F41 strains circulating in Yantai show marked genetic diversity. Notably, HAdV-F41 undergoes significant recombination events that may enhance viral adaptability and immune escape. These findings provide important molecular evidence to inform future surveillance strategies and vaccine antigen design for HAdV-F subgroups.

## 1. Introduction

Human enteric adenovirus (HAdV) is a major etiological agent of diarrheal disease worldwide, with particularly high incidence among children [1]. In developing countries, species F adenoviruses, specifically HAdV-F40 and HAdV-F41, are widely recognized as key viral pathogens responsible for acute gastroenteritis in pediatric populations [2,3]. Clinical manifestations typically include diarrhea, vomiting, low-grade fever, and mild dehydration. Notably, infections caused by HAdV-F40 and HAdV-F41 are often associated with prolonged episodes of diarrhea [3,4].

In recent years, research interest in HAdV-F has grown globally [2]. A study in Kolkata, India, detected HAdV-F41 in 1053 fecal samples from children with diarrhea, highlighting both high prevalence and extensive genetic diversity [4]. Similar findings from Tunisia, Brazil, and Kenya further underscore the widespread distribution and genomic variability of enteric adenoviruses [2,3,5]. In China, HAdV-F41 has also demonstrated significant genomic diversity, with prevalence varying across different years and demographic groups [1]. Collectively, these findings suggest that human adenovirus F subgroups possess substantial global genetic heterogeneity, presenting ongoing challenges for public health surveillance and vaccine development [6].

Despite progress in global epidemiological and genomic studies, data on the regional circulation of human adenovirus F subgroups (HAdV-F40 and F41) within China, particularly in coastal cities like Yantai, remain limited. This study seeks to address this gap by analyzing the genomic epidemiology of HAdV-F40 and HAdV-F41 in Yantai from August 2022 to August 2023 using whole-genome sequencing. The findings aim to provide scientific evidence to inform future strategies for disease prevention, control, and vaccine design.

## 2. Materials and Methods

### 2.1. Sample Collection

Between August 2022 and August 2023, 1200 stool specimens were collected from patients with suspected viral diarrhea in Yantai City, Shandong Province, China, by the Yantai Center for Disease Control and Prevention. All enrolled cases met the clinical and laboratory diagnostic criteria defined in the Diagnostic Criteria for Infectious Diarrhea. The study was reviewed and approved by the Ethics Committee of Preventive Medicine at the Yantai Center for Disease Control and Prevention (approval number: YYLLS 2023-30). Written informed consent was obtained from the legal guardians of all participants.

### 2.2. Nucleic Acid Extraction

Approximately 0.5 g of formed stool, or 50–100 μL of watery feces, was diluted in 500 μL of isotonic sodium chloride solution to prepare a 10–20% suspension. The suspension was centrifuged at 8000 rpm for 5 min. Subsequently, 200 μL of the supernatant was used for nucleic acid extraction, performed with the QIAamp MinElute Virus Spin Kit (QIAGEN, Hilden, Germany) according to the manufacturer’s instructions. Extracted nucleic acids were stored at −70 °C to prevent degradation caused by repeated freeze–thaw cycles.

### 2.3. Screening for Human Adenovirus F Subgroups

Fluorescent PCR amplification was performed using an enteric adenovirus detection kit (Shanghai Berger Medical Technology Co., Ltd., Shanghai, China) specific for HAdV-F subgroups. Samples exhibiting typical amplification curves within the threshold cycle range were considered positive for HAdV-F subgroups. Each screening batch included both positive and negative control samples to ensure the accuracy and reliability of the assay results.

### 2.4. Serotyping of Human Adenovirus F Subgroups

A total of 31 full-genome sequences (>30 kb) of HAdV-F40 and 60 full-genome sequences (>30 kb) of HAdV-F41 were retrieved from the GenBank database. Using reference genomes for HAdV-F (NC_001454), HAdV-F40 (L19443.1), and a region-specific reference for HAdV-F41 (DQ315364.2), serotype-specific primers were designed for both HAdV-F40 and HAdV-F41. Fluorescent PCR was subsequently used to identify and differentiate these serotypes in adenovirus-positive fecal samples.

### 2.5. DNA Sequencing

To increase the likelihood of obtaining complete genome sequences, samples with higher viral loads were selected for sequencing. Whole-genome amplification of HAdV-F40 and HAdV-F41 was performed using serotype-specific enrichment kits targeting adenovirus type 40 and type 41. PCR amplification products were purified using the QIAquick PCR Purification Kit (QIAGEN, Hilden, Germany). Library preparation was carried out using the Illumina Library Preparation Kit (Illumina, San Diego, CA, USA), followed by paired-end sequencing on the Illumina platform.

### 2.6. Data Analysis

Single Nucleotide Polymorphism (SNP) Identification and Phylogenetic Analysis: Reference genomes used for alignment included HAdV-F (NC_001454), HAdV-F40 (L19443.1), and HAdV-F41 (DQ315364.2). Whole-genome assembly for HAdV-F40 and HAdV-F41 was conducted using CLC Genomics Workbench (QIAGEN, Germany). Genomes with >95% coverage were aligned against their respective reference sequences using BLASTN v2.13.0 (NCBI, Bethesda, MD, USA) Multiple sequence alignment was performed with MAFFT, and phylogenetic trees were constructed using MEGA7 software (Version 10.2.6, Pennsylvania State University, State College, PA, USA) based on the General Time Reversible (GTR) model. Tree robustness was assessed via 1000 bootstrap replicates. Genotypic classification and phylogenetic clustering were determined by a combination of the Neighbor-Joining (NJ) method and BLASTN results.

Recombination Analysis: Recombination events in HAdV-F40 and HAdV-F41 were systematically investigated using RDP5 and SimPlot v3.5.1 (Stuart Ray, Johns Hopkins University, Baltimore, MD, USA). Aligned full-genome sequences were analyzed with multiple algorithms in RDP5, including RDP, GENECONV, Bootscan, MaxChi, Chimaera, and SiScan, to identify potential recombination signals. Analyses were performed using a window size of 500 bp and a step size of 100 bp, providing high-resolution detection of recombination breakpoints. Recombination events with *p* < 0.05 were considered statistically significant. SimPlot was used to calculate sequence similarity based on the Jukes-Cantor model and to generate both similarity and bootscan plots.

Structural Modeling: Three-dimensional structural predictions of viral proteins were performed using AlphaFold3 (DeepMind Technologies, London, UK) for both recombinant and reference strains to assess the potential impact of recombination on protein conformation and antigenic characteristics.

## 3. Results

### 3.1. Basic Characteristics of the Study Population

**Figure 1:**
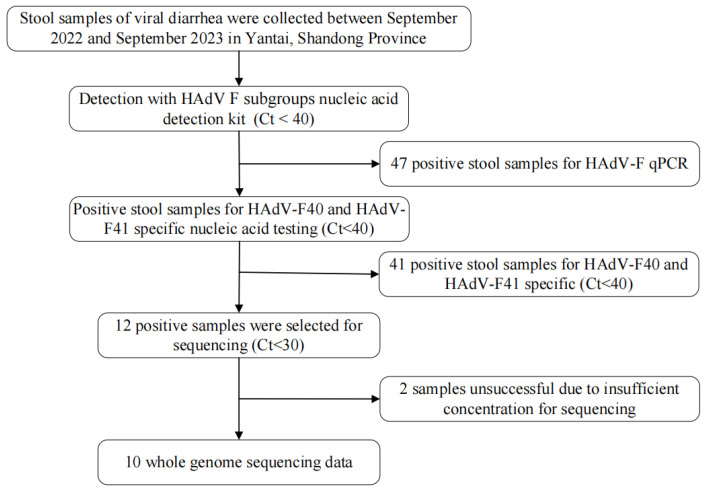
Detection and sample screening workflow of HAdV-F40 and F41 cases in Yantai.

Between 2022 and 2023, a total of 47 HAdV-F positive samples were detected in Yantai City using a nucleic acid detection kit specific for HAdV-F subgroups in diarrheal pathogens (Figure 1). Of these, 5 samples (10.6%) were identified as HAdV-F40, while 36 samples (76.6%) were classified as HAdV-F41.

**Figure 2:**
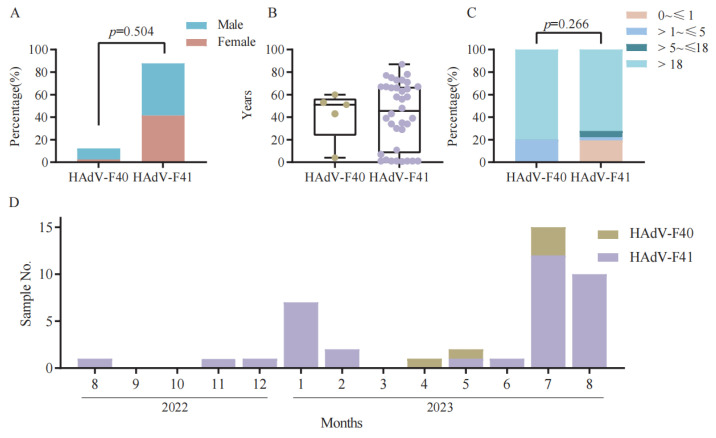
Demographic and seasonal characteristics of HAdV-F40 and HAdV-F41 positive cases in Yantai, China, 2022–2023. (**A**) Sex distribution of patients with HAdV-F40 and HAdV-F41 infections. (**B**) Median age of patients infected with HAdV-F40 and HAdV-F41. (**C**) Distribution of HAdV-F40 and HAdV-F41 positive cases among different age groups (0~≤1, >1~≤5, >5~≤18, and >18 years). (**D**) Monthly distribution of HAdV-F40 and HAdV-F41 positive cases from 2022 to 2023.

The demographic characteristics of the 41 HAdV-F40 and HAdV-F41 positive cases are summarized in Figure 2A–C. Among these cases, 18 were female (43.9%) and 23 were male (56.1%). Both HAdV-F40 and HAdV-F41 infections were predominantly observed in males, although no significant difference was found between the two serotypes (*p* = 0.504). The median age of all patients was 48.0 years (interquartile range [IQR]: 11.00–66.00). Specifically, the median age of patients infected with HAdV-F40 was 51.0 years (IQR: 43.00–53.00), while that of HAdV-F41 cases was 45.5 years (IQR: 10.00–67.00). When grouped by age (0~≤1, >1~≤5, >5~≤18, and >18 years), infections were distributed across all age ranges without statistically significant differences between groups (*p* = 0.266). These findings indicate that HAdV-F subgroups infection is not restricted to pediatric populations but also affects adolescents and adults across a broad age range. For HAdV-F40, cases were mainly concentrated in the >1~≤5 years and >18 years groups, whereas HAdV-F41 infections were more common in the 0~≤1 year and >18 years groups.

The seasonal distribution of 41 positive cases is shown in Figure 2D. Most cases were recorded during the summer months, particularly in July and August, consistent with the typical seasonal pattern of HAdV-F subgroups transmission. Only a few cases were detected during winter and spring months (e.g., January, February, and March), suggesting that elevated temperatures may contribute to increased viral circulation during warmer periods.

### 3.2. Sequencing Analysis of Clinical Samples

All 47 HAdV-F positive samples were subjected to serotype-specific fluorescent PCR targeting HAdV-F40 and HAdV-F41, confirming the presence of both serotypes. Cycle threshold (Ct) values for HAdV-F40 ranged from 13.28 to 36.93, with a median value of 34.84. For HAdV-F41, Ct values ranged from 13.05 to 37.59, with a median value of 33.84.

To ensure high-quality sequencing and optimal genome coverage, samples with Ct values below 30 were selected for whole-genome sequencing. Following sequencing and subsequent analysis, a total of 10 complete and valid HAdV genome sequences were obtained, comprising 1 HAdV-F40 genome and 9 HAdV-F41 genomes.

### 3.3. Single Nucleotide Variants (SNVs) Analysis

Using the reference sequences HAdV-F40 (L19443.1) and HAdV-F41 (DQ315364.2), this study systematically analyzed single nucleotide variants (SNVs) in the three major structural protein-coding genes-*penton*, *hexon*, and *fiber*-of both HAdV-F40 and HAdV-F41 (Table 1).

In the sole HAdV-F40 strain obtained (HAdV-229-Yantai), 8, 16, and 36 SNVs were identified in the *penton*, *hexon*, and *fiber* genes, respectively. These findings indicate a relatively high degree of genetic variability within structural genes, particularly in the *fiber* gene.

In contrast, the nine complete HAdV-F41 genomes exhibited notable differences in SNV distribution across the same gene regions. The *hexon* gene showed the highest mutation density. Specifically, four samples (HAdV-5-Yantai, HAdV-72-Yantai, HAdV-432-Yantai, and HAdV-518-Yantai) harbored 73–77 SNVs, whereas two samples (HAdV-70-Yantai and HAdV-557-Yantai) showed only 15 SNVs. The remaining three samples (HAdV-2-Yantai, HAdV-14-Yantai, and HAdV-322-Yantai) exhibited an intermediate level of mutation (17 SNVs). These differences in mutation burden suggest the presence of multiple genetic variation patterns within the *hexon* region.

For the *fiber* gene, SNV counts in HAdV-F41 ranged from 23 to 34. Three samples (HAdV-2-Yantai, HAdV-14-Yantai, and HAdV-322-Yantai) exhibited higher mutation levels (33–34 SNVs), while the remaining six samples had lower SNV counts (23–27). The *penton* gene demonstrated the greatest sequence conservation across all samples, with only 1 to 4 SNVs identified.

In summary, HAdV-F40 showed the greatest accumulation of mutations in the *fiber* gene, whereas HAdV-F41 displayed pronounced hierarchical variation in the *hexon* gene. These findings highlight the distinct mutational landscapes of the two subtypes and suggest that the *hexon* and *fiber* genes may represent critical targets for viral adaptation and immune evasion.

### 3.4. Phylogenetic Analysis of HAdV-F40/41 Strains

**Figure 3:**
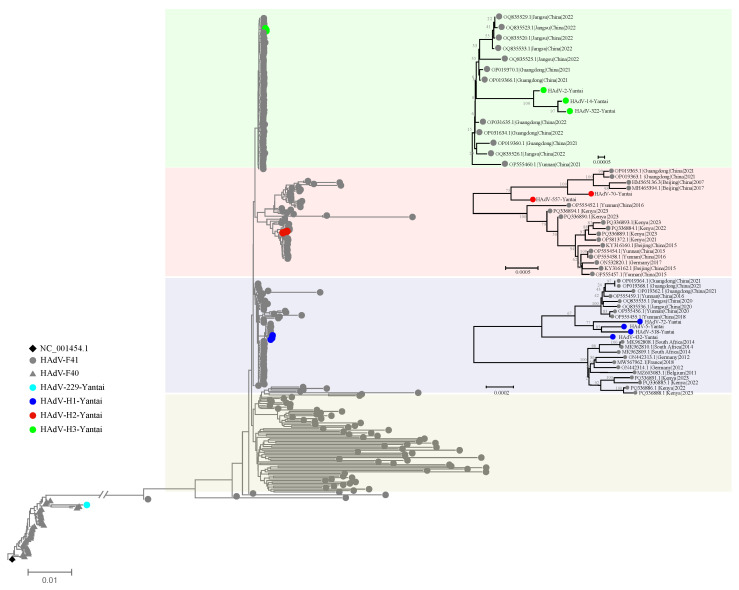
Phylogenetic Tree of HAdV-F40 and F41 Strains. The phylogenetic tree was constructed using the Neighbor-Joining (NJ) method based on 10 full-genome sequences with >95% coverage, combined with reference strain sequences.

A phylogenetic tree was constructed using the Neighbor-Joining (NJ) method based on 10 complete genome sequences with >95% coverage and representative reference sequences retrieved from the NCBI database (Figure 3).

The HAdV-F40 strain (HAdV-229-Yantai) clustered closely with the reference strain NC_001454.1, supported by a high bootstrap value. This indicates that the isolate belongs to the canonical HAdV-F40 lineage and exhibits minimal phylogenetic divergence.

In contrast, the nine HAdV-F41 genome sequences demonstrated evident genetic differentiation within the local population and could be grouped into three distinct clusters:

Cluster H1: Comprising HAdV-5-Yantai, HAdV-72-Yantai, HAdV-432-Yantai, and HAdV-518-Yantai, this group formed a well-supported branch and showed the highest mutation burden in the *hexon* gene, as confirmed by SNV analysis (Table 1).

In addition to the Yantai isolates, Cluster H1 also included reference strains from Guangdong (China), South Africa, Germany, France, Belgium, and Kenya, suggesting a globally distributed lineage with broad genetic connectivity.

Cluster H2: Including HAdV-70-Yantai and HAdV-557-Yantai, this branch was genetically distinct and exhibited the lowest number of SNVs across all three structural genes.

Cluster H2 was closely related to reference strains from Guangdong, Beijing, and Yunnan (China), as well as Kenya and Germany, indicating regional linkage.

Cluster H3: Composed of HAdV-2-Yantai, HAdV-14-Yantai, and HAdV-322-Yantai, this independent lineage displayed intermediate levels of *hexon* gene variation but the highest SNV counts in the *fiber* gene among all HAdV-F41 samples.

Cluster H3 showed close genetic relationships with reference isolates from Jiangsu, Guangdong, and Yunnan (China), suggesting that this lineage may represent a regionally circulating variant within China.

Beyond the three locally circulating clusters identified in Yantai, an additional major subgroup, termed Cluster H4, was recognized as a globally distributed lineage of HAdV-F41. The widespread presence of this cluster underscores its evolutionary success and its value as a key marker for tracing the global transmission and molecular epidemiology of HAdV-F41.

Overall, the phylogenetic clustering patterns closely mirrored the SNV distribution summarized in Table 1. These results suggest that the observed genetic diversification of HAdV-F41 strains is strongly associated with mutation load in key structural protein-coding regions. The differential variation in the *hexon* and *fiber* genes among the subgroups likely reflects distinct adaptive pressures and evolutionary dynamics during local transmission.

### 3.5. Recombination Analysis of HAdV-F40 and F41

No significant intra-typic recombination events were detected in the HAdV-F40 genome. Phylogenetic and sequence alignment analyses indicated that the observed genetic diversity of the HAdV-F40 strain isolated in Yantai was primarily driven by point mutations, suggesting that this subtype maintains a relatively high level of genetic stability and conservation within the region.

**Figure 4:**
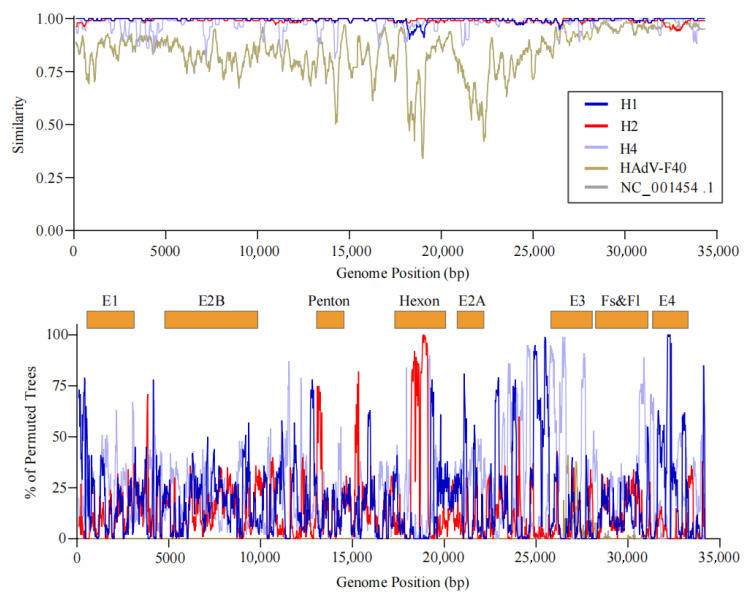
Recombination analysis of HAdV-F subgroups. This figure presents the recombination analysis of the full genome of HAdV-F subgroups using SimPlot and Bootscan methods. The upper panel shows the similarity plot, illustrating sequence identity of group H1, group H2, group H4 and HAdV-F40 relative to the recombinant strain group H3, with potential recombination breakpoints marked. The lower panel displays the Bootscan plot, showing the percentage of phylogenetic support for recombination events across different genomic regions. The x-axis indicates the major functional regions of the adenovirus genome (including E1, E2B, *penton*, *hexon*, E2A, E3, Fs & Fl, and E4), providing a comprehensive view of recombination hotspots.

In contrast, HAdV-F41 exhibited clear evidence of recombination. Specifically, the H3 subgroup strains were identified as potential recombinant lineages, likely arising from genetic fragment exchange between H1 subgroup strains (major donors) and H2 subgroup strains (minor donors). Recombination breakpoints were predominantly located within the *hexon* gene region, further supporting the occurrence of intra-typic recombination events among circulating HAdV-F41 strains (Figure 4).

### 3.6. Structural Prediction Analysis

**Figure 5:**
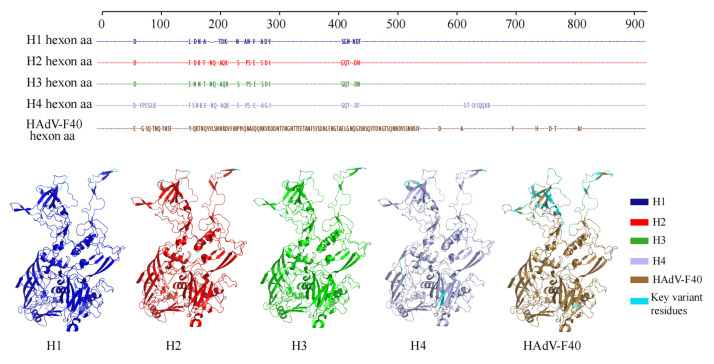
Comparative structural modeling and sequence of *hexon* proteins from different HAdV-F subgroups. *hexon* proteins from four HAdV-F41 subgroups (H1-H4) and HAdV-F40 were modeled using the AlphaFold3 platform. Sequence alignments of the *hexon* amino acid residues are shown in blue (H1), red (H2), green (H3), purple (H4), and brown (HAdV-F40). The corresponding AlphaFold-predicted 3D structures are displayed below, with key variable or divergent amino acid residues among the clusters highlighted in light blue. The root mean square deviations (RMSDs) between representative HAdV-F41 subgroups were 0.144 Å (H3 vs. H1), 0.175 Å (H3 vs. H2), 0.184Å (H3 vs. H4) and 0.157Å (H3 vs. HAdV-F40), indicating high structural similarity among subgroups.

To evaluate the potential impact of recombination and genetic divergence on viral protein structure, three-dimensional modeling of the *hexon* protein from different HAdV-F subgroups was performed using the AlphaFold3 platform (Figure 5).

The analysis revealed a high degree of structural consistency among the *hexon* proteins of the H1, H2, and H3 subgroups. Root-mean-square deviation (RMSD) values calculated based on Cα atoms were 0.144 Å between H3 and H1, and 0.175 Å between H3 and H2, indicating that the recombination events did not significantly alter the global folding conformation of the protein.

However, notable amino acid substitutions were observed in key antigenic determinant regions. Specifically, the H1 subgroup exhibited multiple amino acid changes within surface loop regions, which may induce local conformational alterations and potentially modulate epitope exposure. In contrast, the H2 subgroup demonstrated greater structural conservation in these regions, reflecting a higher degree of overall stability. The recombinant H3 subgroup showed amino acid substitutions at several positions; although the number of mutations was limited, their location in antigenically important regions warrants attention.

In addition to these locally circulating subgroups, a fourth major lineage, designated as H4, was identified as a globally prevalent HAdV-F41 cluster. Structural modeling revealed that the H4 *hexon* protein maintained the overall architecture observed in the H1–H3 subgroups but contained distinctive amino acid substitutions concentrated in exposed surface regions, particularly within hypervariable loops. These substitutions may reflect adaptive evolution under immune selection pressure, contributing to the successful global dissemination of this lineage.

Overall, these findings suggest that although intra-typic recombination and sequence variation in HAdV-F41 do not disrupt the overall tertiary structure of the *hexon* protein, localized alterations within antigenic regions—including those observed in the globally prevalent H4 lineage—may influence viral antigenicity and immune recognition, thereby enhancing viral adaptability and evolutionary fitness.

## 4. Discussion

This study provides a comprehensive molecular epidemiological overview of human enteric adenovirus types F40 and F41 in Yantai from 2022 to 2023. Through whole-genome sequencing and comparative analysis, we delineated their phylogenetic structures, mutation profiles, and recombination patterns. Our findings show that HAdV-F40 exhibits a relatively conserved genomic architecture, with evolution primarily driven by point mutations. In contrast, HAdV-F41 displays substantial genetic heterogeneity, characterized by clear phylogenetic divergence and intra-typic recombination, resulting in three distinct evolutionary clusters. Structural modeling further revealed that, although recombination did not alter the global conformation of the *hexon* protein, amino acid substitutions were concentrated in key antigenic regions, potentially affecting immune recognition. These results highlight distinct evolutionary trajectories of HAdV-F40 and F41 and provide a molecular basis for understanding their antigenic variability, with important implications for regional surveillance and vaccine design.

From an epidemiological perspective, this study confirms the presence of both HAdV-F40 and HAdV-F41 across a wide age range in the Yantai population, not limited to children. While previous studies from Brazil [2] and India [7] have reported HAdV-F41 as predominantly affecting children under five years of age, our findings reveal that a significant proportion of infections occurred in adolescents and adults. This observation is consistent with findings from Denmark, where adenovirus F40/F41 was detected across various age groups [8], which suggests underrecognized adult infections. This finding highlights the importance of ongoing molecular surveillance across all age groups.

In terms of genetic diversity, this study demonstrated that HAdV-F40 exhibits relatively limited genomic variability compared to HAdV-F41, consistent with observations from other geographic regions [5]. In contrast, HAdV-F41 displayed extensive non-synonymous mutations across multiple lineages, with a marked accumulation in the hypervariable regions of the *hexon* gene [9,10,11]—a pattern similarly reported in molecular studies from India and Brazil [2,7]. Such variability likely reflects viral adaptation under selective immune pressure, potentially facilitating persistent transmission across heterogeneous host populations. Notably, the three distinct phylogenetic clusters identified in this study (H1, H2, H3) exhibited not only consistent genomic divergence but also stable lineage-specific topologies within the phylogenetic tree, suggesting that they may represent regionally circulating evolutionary trajectories with potential public health relevance.

Recombination analysis provides critical insights into the evolutionary trajectory of HAdV-F41. This study identified that gene fragments within H3 recombinant strains could be traced to distinct subgroups, aligning with genomic surveillance data from Beijing (2010–2019), which reported multiple recombination events within HAdV-F41 [1]. While frequent recombination has been well-documented as a key evolutionary mechanism in adenovirus species B, C, and D, F40 and F41 were historically regarded as more conserved [12]. However, accumulating evidence in recent years has indicated that recombination events are occurring within these lineages [10,13,14]. The findings of this study further support the notion that recombination is not only a major contributor to the genetic diversity of HAdV-F41 but may also play a role in enhancing viral adaptability during localized outbreaks and cross-regional transmission.

From a structural perspective, AlphaFold3-based modeling in this study revealed that recombination did not alter the global folding conformation of the *hexon* protein, as reflected by consistently low RMSD values across subgroups. However, within key antigenic determinant regions—particularly the surface loop domains—multiple amino acid substitutions were identified in both H1 and H3 strains, suggesting potential local conformational changes that may impair the binding efficiency of neutralizing antibodies [15,16,17,18]. A similar pattern was reported in pediatric samples from Kolkata, India (2007–2009), where emerging HAdV-F41 strains accumulated critical mutations within the hypervariable regions of the *hexon* gene and demonstrated distinctive clinical profiles [4]. These localized structural variations, as observed in the present study, may hold functional relevance and offer valuable insights for future vaccine antigen design and immunogenic target optimization.

This study has several limitations. First, the relatively small sample size and the limited number of successfully sequenced samples have led to a scarcity of whole genome sequences (only 10 cases), which may affect the general applicability of the research results and may also underestimate the true extent of genetic diversity and recombination frequency within the prevalent strains. Second, while structural predictions offer valuable preliminary insights, the functional consequences of recombination events and antigenic variation remain to be experimentally validated—such as through neutralization assays or immune escape studies.

Future research should involve large-scale, multicenter whole-genome surveillance encompassing diverse geographic regions and age groups, integrated with serological and immunological investigations, to clarify the biological relevance of the observed mutations and recombination patterns. In addition, future studies involving cell-based neutralization assays and epitope mapping using representative HAdV-F41 subtype strains are planned to experimentally validate the predicted impact of recombination on viral antigenicity. These efforts will help to further substantiate the molecular and immunological implications inferred from the current genomic analyses.

In summary, this study provides a comprehensive genomic characterization of HAdV-F40 and HAdV-F41 circulating in the Yantai region, revealing marked genetic diversity and lineage-specific recombination within HAdV-F41. Structural analysis further highlights the potential immunological implications of amino acid substitutions in key antigenic regions. These findings advance the understanding of human adenovirus F subgroups evolution and offer important insights for public health surveillance and the rational design of future vaccines.

## Figures and Tables

**Table 1:** Distribution of Identified SNPs in the Whole Genomes of Two Adenovirus Subtypes.

ID	Cluster	Subtype	*Penton* Gene	*Hexon* Gene	*Fiber* Gene
HAdV-229-Yantai	/	HAdV-F40 ^a^	8	16	36
HAdV-5-Yantai	HAdV-H1	HAdV-F41 ^b^	3	77	26
HAdV-72-Yantai	HAdV-H1	HAdV-F41	3	74	26
HAdV-432-Yantai	HAdV-H1	HAdV-F41	3	73	27
HAdV-518-Yantai	HAdV-H1	HAdV-F41	3	77	26
HAdV-70-Yantai	HAdV-H2	HAdV-F41	1	15	23
HAdV-557-Yantai	HAdV-H2	HAdV-F41	1	15	23
HAdV-2-Yantai	HAdV-H3	HAdV-F41	4	17	33
HAdV-14-Yantai	HAdV-H3	HAdV-F41	4	17	34
HAdV-322-Yantai	HAdV-H3	HAdV-F41	4	17	34

^a^ Reference sequence: L19443.1; ^b^ Reference sequence: DQ315364.2.

## Data Availability

All available data regarding this manuscript is presented in the present manuscript.
